# Protein Redox State Monitoring Studies of Thiol Reactivity

**DOI:** 10.3390/antiox8050143

**Published:** 2019-05-22

**Authors:** Yuichiro J. Suzuki, Lucia Marcocci, Takashi Shimomura, Yuki Tatenaka, Yuya Ohuchi, Tinatin I. Brelidze

**Affiliations:** 1 Department of Pharmacology and Physiology, Georgetown University Medical Center, Washington, DC 20057, USA; 2 Department of Biochemical Sciences “A. Rossi Fanelli”, Sapienza University of Rome, 00185 Rome, Italy; Lucia.Marcocci@uniroma1.it; 3 Dojindo Laboratories, 2025-5 Tabaru, Mashiki-machi, Kumamoto 861-2202, Japan; t-shimomura@dojindo.co.jp (T.S.); y-tatenaka@dojindo.co.jp (Y.T.); ouchi@dojindo.co.jp (Y.O.)

**Keywords:** cysteine, HCN channel, hydrogen peroxide, peroxiredoxin, protein, redox state

## Abstract

Protein cysteine thiol status is a major determinant of oxidative stress and oxidant signaling. The -*SulfoBiotics*- Protein Redox State Monitoring Kit provides a unique opportunity to investigate protein thiol states. This system adds a 15-kDa Protein-SHifter to reduced cysteine residues, and this molecular mass shift can be detected by gel electrophoresis. Even in biological samples, Protein-SHifter Plus allows the thiol states of specific proteins to be studied using Western blotting. Peroxiredoxin 6 (Prx6) is a unique one-cysteine peroxiredoxin that scavenges peroxides by utilizing conserved Cysteine-47. Human Prx6 also contains an additional non-conserved cysteine residue, while rat Prx6 only has the catalytic cysteine. In cultured cells, cysteine residues of Prx6 were found to be predominantly fully reduced. The treatment of human cells with hydrogen peroxide (H_2_O_2_) formed Prx6 with one cysteine reduced. Since catalytic cysteine becomes oxidized in rat cells by the same H_2_O_2_ treatment and treating denatured human Prx6 with H_2_O_2_ results in the oxidation of both cysteines, non-conserved cysteine may not be accessible to H_2_O_2_ in human cells. We also found that untreated cells contained Prx6 multimers bound through disulfide bonds. Surprisingly, treating cells with H_2_O_2_ eliminated these Prx6 multimers. In contrast, treating cell lysates with H_2_O_2_ promoted the formation of Prx6 multimers. Similarly, treating purified preparations of the recombinant cyclic nucleotide-binding domain of the human hyperpolarization-activated cyclic nucleotide-modulated channels with H_2_O_2_ promoted the formation of multimers. These studies revealed that the cellular environment defines the susceptibility of protein cysteines to H_2_O_2_ and determines whether H_2_O_2_ acts as a facilitator or a disrupter of disulfide bonds.

## 1. Introduction

The redox state of protein cysteine residues plays a crucial role in cell regulation and is often the major target of oxidative stress and oxidant signaling conferred by reactive oxygen species [1,2,3,4,5,6,7]. Various labeling probes allow for the determination of the global protein thiol status in the cells [8]. However, the examination of the thiol states of specific cellular proteins has been difficult. In this regard, Dojindo Molecular Technologies, Inc. has developed a novel approach that allows easy determination of the cysteine redox states within the proteins of interest. In the -*SulfoBiotics*- Protein Redox State Monitoring Kit system, a 15-kDa Protein-SHifter is added to every reduced cysteine residue, allowing for the defining of protein redox states using gel electrophoresis while examining the mobility shift. Furthermore, in the improved “Plus” kit, the bulky Protein-SHifter is eliminated by UV light after gel electrophoresis, making the system suitable for Western blotting. Here, we investigated the capability of the -*SulfoBiotics*- Protein Redox State Monitoring Kit to report on the cysteine redox state of two structurally and functionally unrelated human proteins, Prx6 and cyclic nucleotide-binding domain (CNBD) of HCN4 channels, one of which (CNBD) we have the ability to purify with affinity and size-exclusion chromatography.

Peroxiredoxins are a class of peroxidase antioxidant enzymes that play critical roles in regulating redox homeostasis [9,10]. Peroxiredoxin 1 was first discovered by Su Goo Rhee, Earl Stadtman, and co-workers [11], and six members of peroxiredoxins have so far been identified [9,10,12]. Peroxiredoxins 1–5 were found to contain two catalytic cysteines that serve as electron donors to perform the two-electron reduction of H_2_O_2_ to produce H_2_O [9,10]. Peroxiredoxin 6 (Prx6) is unique in that this protein possesses only one cysteine, which serves as the catalytic cysteine. It is thought that the second electron for the two-electron reduction is donated by glutathione [13,14].

Hyperpolarization-activated cyclic nucleotide-modulated (HCN) channels belong to the super-family of voltage-gated potassium channels and generate rhythmic activity of neurons and cardiomyocytes [15]. HCN channels contain cyclic nucleotide-binding domains (CNBD) in their C-terminal region, which regulate the activity of HCN channels through cyclic nucleotide binding [16]. There are four HCN channel isoforms in humans (HCN1–HCN4) with distinct cyclic nucleotide sensitivity [15]. The HCN channels have been extensively studied with electrophysiology, fluorescence-based, and structural methods, however, few studies have addressed the redox state of cysteines in these channels.

The present study was designed to use the -*SulfoBiotics*- Protein Redox State Monitoring system to understand the redox behaviors of cysteine residues of two structurally unrelated proteins, Prx6 in human vascular smooth muscle cells and the purified CNBD domain of the human HCN4 channel.

## 2. Methods

### 2.1. Protein Expression and Purification

CNBD of human HCN4 (hHCN4) (amino acids 571–708, gene identifier Q9Y3Q4) were subcloned into a pETM11 bacterial expression vector containing an N-terminal 6-His affinity tag. The proteins were expressed in BL21 (DE3) cells and purified with Ni^2+^–NTA and size-exclusion chromatography, as previously described [17]. The final purification step was done in 150 mM KCl, 10% glycerol, 1 mM tris(2-carboxyethyl)phosphine (TCEP), 30 mM HEPES; pH 7.5. The molecular weight of the purified protein was verified with mass spectrometry (electrospray) at the Georgetown University Proteomics and Metabolomics Shared Resources core facility. The purified protein was stored at −80 °C in small aliquots before use.

### 2.2. Cell Culture

Human pulmonary artery smooth muscle cells were purchased from ScienCell Research Laboratories (Carlsbad, CA, USA), and rat pulmonary artery smooth muscle cells were purchased from Cell Applications (San Diego, CA, USA). Cells were cultured in accordance with the manufacturers’ instructions in 5% CO_2_ at 37 °C. Cells were treated with H_2_O_2_ purchased from Sigma-Aldrich Chemical Company (St. Louis, MO, USA).

### 2.3. Protein Redox State Monitoring

Protein thiol states were monitored using the -*SulfoBiotics*- Protein Redox State Monitoring Kit (Catalog #SB11) for purified recombinant proteins and the -*SulfoBiotics*- Protein Redox State Monitoring Kit Plus (Catalog #SB12) for cellular proteins in accordance with the manufacturer’s instructions (Dojindo Molecular Technologies Inc., Rockville, MD, USA).

To study the redox states in the purified recombinant proteins, samples were subjected to non-reducing sodium dodecyl sulfate (SDS) polyacrylamide gel electrophoresis (PAGE) and visualized by Coomassie Brilliant Blue staining.

To study the protein redox states in the cultured cells, samples were prepared through protein precipitation using trichloroacetic acid (TCA; VWR International, Radnor, PA, USA) and labeled with Protein-SHifter Plus in accordance with the manufacturer’s instructions for the -*SulfoBiotics*- Protein Redox State Monitoring Kit Plus. After cell extracts were subjected to SDS-PAGE, gels were exposed to UV light on a transilluminator to remove Protein-SHifter. Proteins in the gel were then electro-transferred to a nitrocellulose membrane (Bio-Rad Laboratories, Hercules, CA, USA). The membrane was blocked with 5% non-fat dry milk and incubated with rabbit Prx6 IgG antibody (Sigma-Aldrich). Signals were obtained by using a horseradish peroxidase-linked secondary antibody (Bio-Rad) and the Enhanced Chemiluminescence System (GE Healthcare Bio-Sciences, Pittsburgh, PA, USA) and were computed for Western blot band intensities. Protein concentrations of cell lysates were measured using the bicinchoninic acid (BCA) assay (Pierce BCA Protein Assay Kit, Thermo Fisher Scientific, Waltham, MA, USA).

### 2.4. Statistical Analysis

Means and standard errors of mean (SEM) were computed. Two groups were compared by a two-tailed Student’s *t* test, and differences between more than two groups were determined by the analysis of variance (ANOVA). *p* < 0.05 was defined to be statistically significant.

## 3. Results

### 3.1. -SulfoBiotics- Protein Redox State Monitoring Kit to Assess Thiol States of Purified Proteins

**Figure 1:**
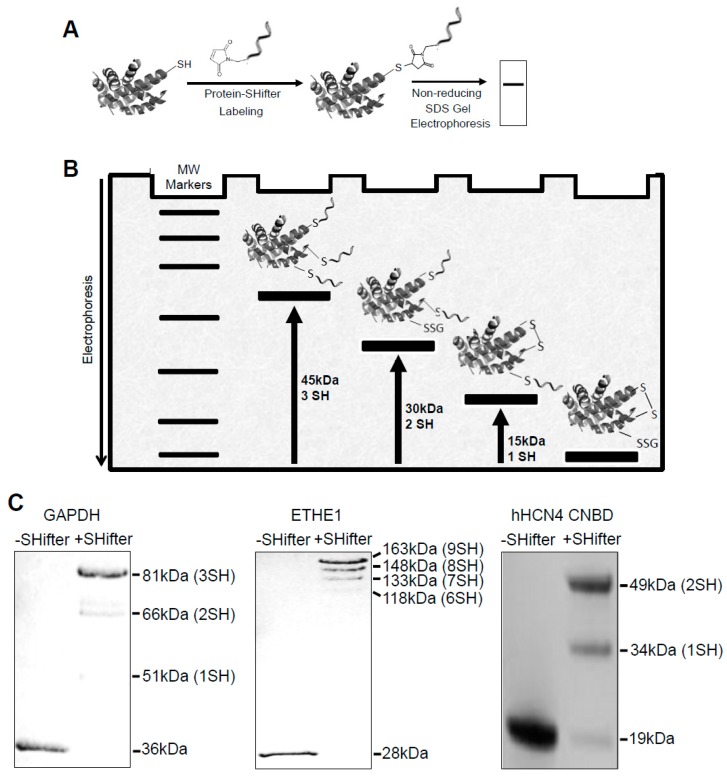
-*SulfoBiotics*- Protein Redox State Monitoring Kit. (**A**) The principle of the -*SulfoBiotics*- Protein Redox State Monitoring Kit. A free protein thiol group was labeled with the Protein-SHifter that contained maleimide with a high affinity toward reduced cysteine. Samples were then electrophoresed and stained. (**B**) For each Protein-SHifter bound to a protein molecule, a 15-kDa shift was observed in the electrophoresis. (**C**) Examples of the use of the -*SulfoBiotics*- Protein Redox State Monitoring Kit to determine the thiol redox state of recombinant proteins.

The -*SulfoBiotics*- Protein Redox State Monitoring Kit allows the detection of reduced protein cysteine residues by labeling with Protein-SHifter composed of a maleimide group that covalently binds to a protein thiol group and a DNA moiety that has a molecular mass of ~15 kDa [18,19]. Labeled protein samples are subjected to non-reducing SDS-PAGE and visualized by staining the gel (Figure 1A). If the protein contains one reduced cysteine, the electrophoretic band will be shifted by 15 kDa upward. If the protein contains two reduced cysteines, two Protein-SHifters bind to the protein molecule, causing a total of a 30 kDa shift as described in the schematics of Figure 1B.

Figure 1C shows examples of how reduced cysteines may be detected in purified recombinant proteins. With Protein-SHifter, glyceraldehyde-3-phosphate dehydrogenase (GAPDH) with a molecular weight of 36 kDa and containing three cysteine residues exhibited ~51, ~66, and ~81 kDa bands, indicating that one, two, or three cysteine residues can be in the reduced state. In this particular preparation, the GAPDH protein molecules with three cysteine residues in the reduced state were the most prominent. The 28-kDa ethylmalonic encephalopathy 1 (ETHE1) protein molecule with nine cysteines was found to be highly reduced in this recombinant protein preparation.

We also tested the effects of Protein-SHifter on the recombinant CNBD domain of hHCN4 channels (19 kDa) with two cysteines. The results showed two bands corresponding to the modifications with one (34-kDa) and two (49-kDa) Protein-SHifter molecules. The observation that the 49-kDa species of the purified CNBD was predominant indicates that both cysteine residues reacted with the Protein-SHifter, which is consistent with the reducing effect of TCEP present in the protein solution.

### 3.2. Redox Studies of Prx6 in Cultured Cells Using -Sulfobiotics- Protein Redox State Monitoring Kit Plus

**Figure 2:**
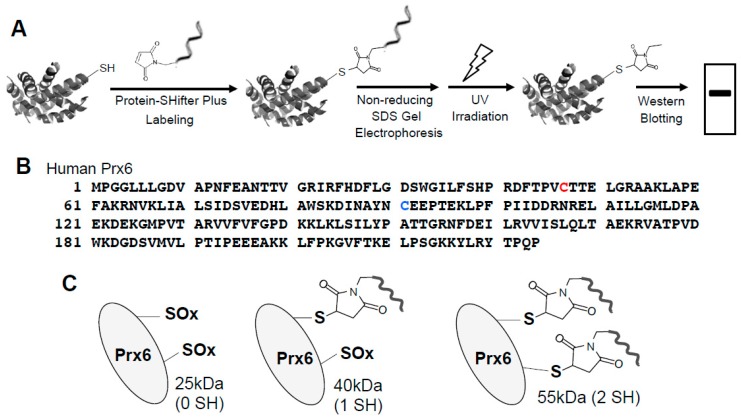

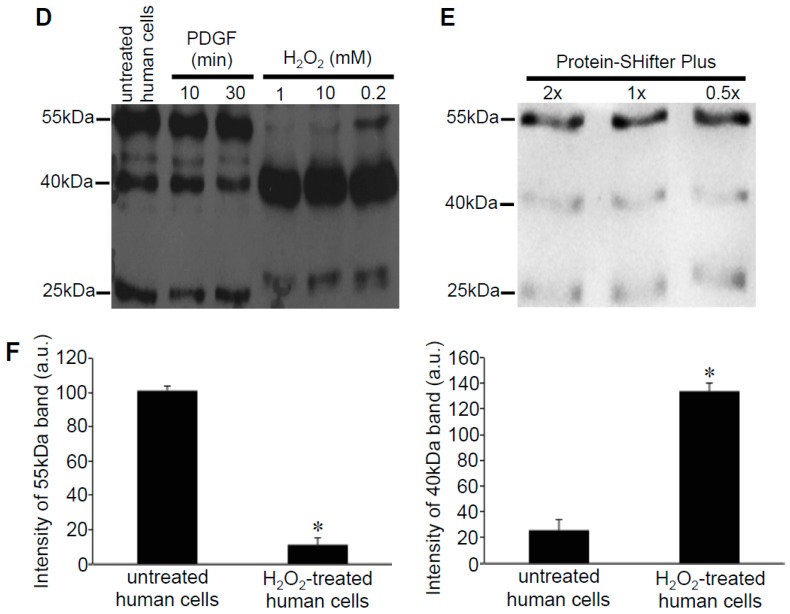
Effects of H_2_O_2_ on thiol state of Prx6 in human cells monitored using Protein-SHifter Plus. (**A**) The principle of the -*SulfoBiotics*- Protein Redox State Monitoring Kit Plus. A free protein thiol group was labeled with the Protein-SHifter Plus that contained maleimide with a high affinity toward reduced cysteine. After electrophoresis, the large Protein-SHifter Plus moiety was eliminated with UV light, increasing the efficiency of Western blotting and allowing for the detection of specific proteins in the biological samples. (**B**) Amino acid sequence of human Prx6. Cys47 indicated in red is conserved catalytic cysteine, while Cys91 indicated in blue is non-conserved cysteine. (**C**) Schematics of the human Prx6 structure with or without Protein-SHifter Plus attached. SOx indicates oxidized cysteine that would not bind to the Protein-SHifter Plus. (**D**) Human pulmonary artery smooth muscle cells were treated with or without PDGF (10 ng/mL) or H_2_O_2_ at indicated concentrations for 15 min. Cell lysates were prepared, incubated with Protein-SHifter Plus, subjected to SDS-PAGE without BME, and immunoblotted with the Prx6 antibody. (**E**) Untreated cell lysates were incubated with different amounts of Protein-SHifter Plus with 1x being the amount used in all experiments in this study. (**F**) Bar graphs represent the means ± SEM of the intensities of 55- and 40-kDa bands in arbitrary unit (a.u.) (*n* = 5). The symbol * represents the value significantly different from the untreated control value at *p* < 0.05.

In biological samples containing multiple proteins, proteins of interest can be studied using the -*SulfoBiotics*- Protein Redox State Monitoring Kit Plus. This “Plus” system contains the 15-kDa Protein-SHifter Plus whose DNA moiety can come off after electrophoresis by exposing the gels to UV light, aiding in visualizing the thiol redox states using Western blotting (Figure 2A) [19].

We treated human smooth muscle cells with either platelet-derived growth factor (PDGF) that may elicit oxidant signaling [20] or H_2_O_2_. Cells were treated with H_2_O_2_ for 15 min in accordance with the product instructions of the -*SulfoBiotics*- Protein Redox State Monitoring Kit Plus. We prepared cell lysates in accordance with the -*SulfoBiotics*- Protein Redox State Monitoring Kit Plus protocol using the cell lysate buffer that was included in the kit after the TCA protein precipitation. We electrophoresed the Protein-SHifter Plus-labeled samples under the non-reducing condition, eliminated the DNA moiety of the Protein-SHifter Plus by UV light and blotted onto a nitrocellulose membrane, followed by immunoblotting with the Prx6 antibody.

The human peroxiredoxin 6 (Prx6) protein has a molecular weight of 25-kDa and contains two cysteine residues: a catalytic cysteine at position 47 responsible for the peroxidase activity and a non-conserved cysteine at position 91 (Figure 2B). Our results showed that after labeling with Protein-SHifter Plus, Prx6 molecules from untreated human smooth muscle cells predominantly contained the 55-kDa species. This was consistent with the protein having two Protein-SHifters incorporated (Figure 2C), suggesting that both of the cysteine residues were mostly in the reduced state in the cells (Figure 2D). The band patterns were not altered by changing the amounts of Protein-SHifter Plus (Figure 2E). While the treatment of cells with PDGF did not influence the thiol state of Prx6, the treatment of cells with H_2_O_2_ as low as 200 μM for 15 min dramatically decreased the 55-kDa band and increased the 40-kDa band (Figure 2D,F), indicating that only one sulfhydryl group was oxidized. This event does not appear to be Fenton reaction-dependent since the pre-treatment of cells with deferoxamine (50 μM) for 30 min did not influence the H_2_O_2_ effects (data not shown).

**Figure 3:**
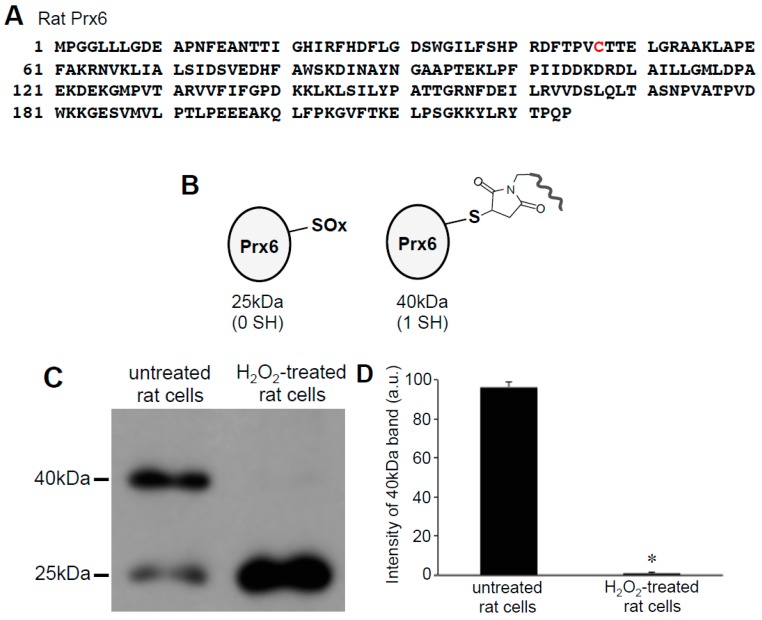
Effects of H_2_O_2_ on thiol state of Prx6 in rat cells monitored using Protein-SHifter Plus. (**A**) Amino acid sequence of rat Prx6. Cys47 indicated in red is the conserved catalytic cysteine. (**B**) Schematics of the rat Prx6 structure with or without Protein-SHifter Plus attached. SOx indicates the oxidized cysteine that would not bind to the Protein-SHifter Plus. (**C**) Rat pulmonary artery smooth muscle cells were treated with or without H_2_O_2_ (200 μM) for 15 min. Cell lysates were prepared, incubated with Protein-SHifter Plus, subjected to SDS-PAGE without BME, and immunoblotted with the Prx6 antibody. (**D**) The bar graph represents the means ± SEM of the intensity of the 40 kDa band (*n* = 3). The symbol * represents the value significantly different from the untreated control value at *p* < 0.05.

Since only one cysteine out of two in human Prx6 was found to be oxidized by H_2_O_2_, we examined rat Prx6, which contains only one cysteine that is the conserved catalytic cysteine at position 47 (Figure 3A). Using cultured rat smooth muscle cells, we performed the same Protein-SHifter Plus experiments as those performed in human cells. Results showed that the untreated rat cells contained Prx6 molecules that predominantly exhibited the 40 kDa band, which was consistent with one cysteine being reduced (Figure 3B), suggesting that the catalytic cysteine was in the reduced state in cells. The treatment of cells with 200 μM H_2_O_2_ for 15 min eliminated the 40 kDa band and produced the 25 kDa band (Figure 3C,D), consistent with the oxidation of the catalytic cysteine by H_2_O_2_.

**Figure 4:**
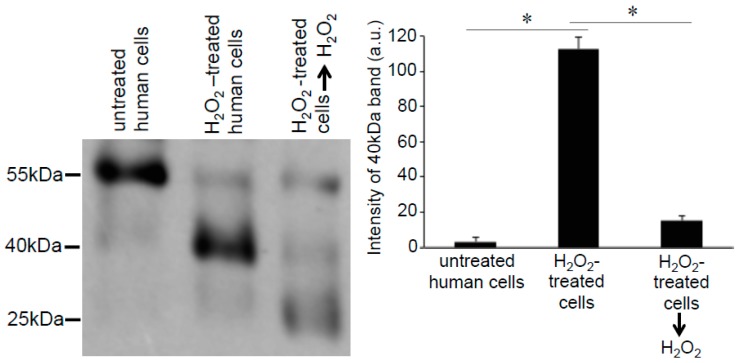
Effects of H_2_O_2_ on thiol state of human Prx6 in cell lysates monitored using Protein-SHifter Plus. Cell lysates were prepared from untreated human pulmonary artery smooth muscle cells (Lane 1) or cells treated with H_2_O_2_ for 15 min (Lane 2). Lysate proteins from H_2_O_2_-treated cells were denatured with SDS and incubated with H_2_O_2_ (1 mM) in a test tube for 15 min (Lane 3). Protein-SHifter Plus was then added and the samples subjected to SDS-PAGE without BME and immunoblotted with the Prx6 antibody. The bar graph represents the means ± SEM of the intensity of the 40 kDa bands (*n* = 3). The symbol * represents the value significantly different from each other at *p* < 0.05.

To test the hypothesis that one cysteine residue of human Prx6 is not accessed by H_2_O_2_ in cells, we treated cell lysates with H_2_O_2_ after the proteins were denatured by SDS and before the addition of Protein-SHifter Plus. In contrast to the cell treatment with H_2_O_2_, which increased the 40 kDa Prx6 band (lane 1 vs. lane 2 in Figure 4), the ex vivo treatment with H_2_O_2_ dramatically decreased the 40 kDa Prx6 band (lane 2 vs. lane 3 in Figure 4). These results suggest that, perhaps due to the three-dimensional structure of Prx6 defined by the cellular environment, oxidation of non-conserved cysteine with H_2_O_2_ is blocked in the human Prx6 protein.

### 3.3. H_2_O_2_ Can Decrease Disulfide Bonds in the Cells

**Figure 5:**
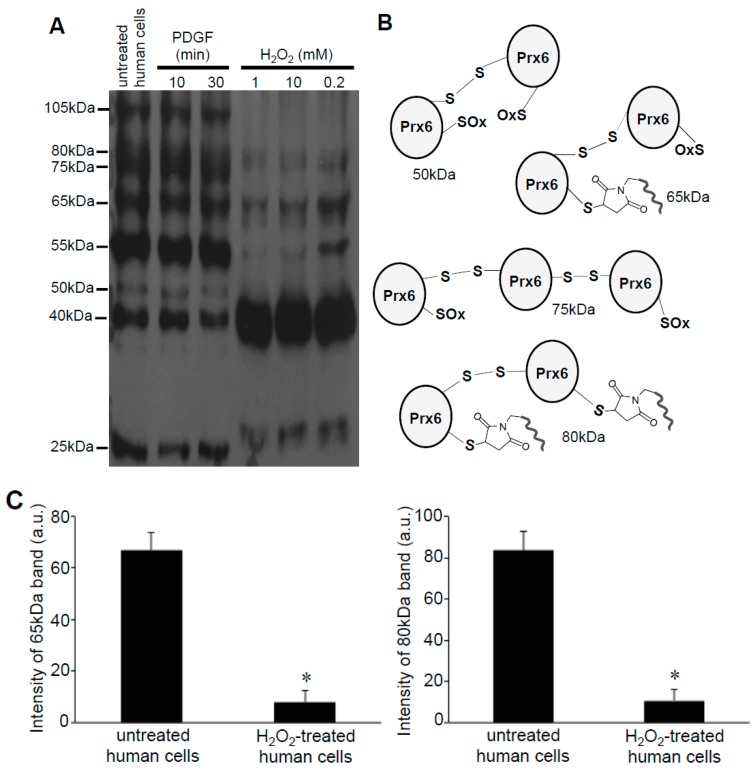
Identification of Prx6 multimers in human cells. (**A**) Human pulmonary artery smooth muscle cells were treated with or without PDGF (10 ng/mL) or H_2_O_2_ at indicated concentrations for 15 min. Cell lysates were prepared, incubated with Protein-SHifter Plus, subjected to SDS-PAGE without BME, and immunoblotted with the Prx6 antibody. (**B**) Possible structures of Prx6 that produce bands of various molecular weights. (**C**) Bar graphs represent the means ± SEM of the intensities of 65- and 80-kDa bands (*n* = 4). The symbol * represents the value significantly different from the untreated control value at *p* < 0.05.

As the -*SulfoBiotics*- Protein Redox State Monitoring Kit Plus requires running non-reducing gels, in addition to the 25-, 40-, and 55-kDa bands that were expected to occur in the Prx6 monomer with or without the Protein SHifter, we also noticed higher molecular weight bands in these gels (Figure 5A). These bands included 50-, 65-, 75-, 80-, and 105-kDa bands consistent with the occurrence of dimers and trimers of various redox states with different numbers of Protein-SHifters interacting with Prx6 (Figure 5B). Interestingly, the treatment of cells with H_2_O_2_ for 15 min resulted in the elimination of these multimers, indicating that H_2_O_2_ may have disrupted the protein disulfide bonds in the cells (Figure 5A,C).

**Figure 6:**
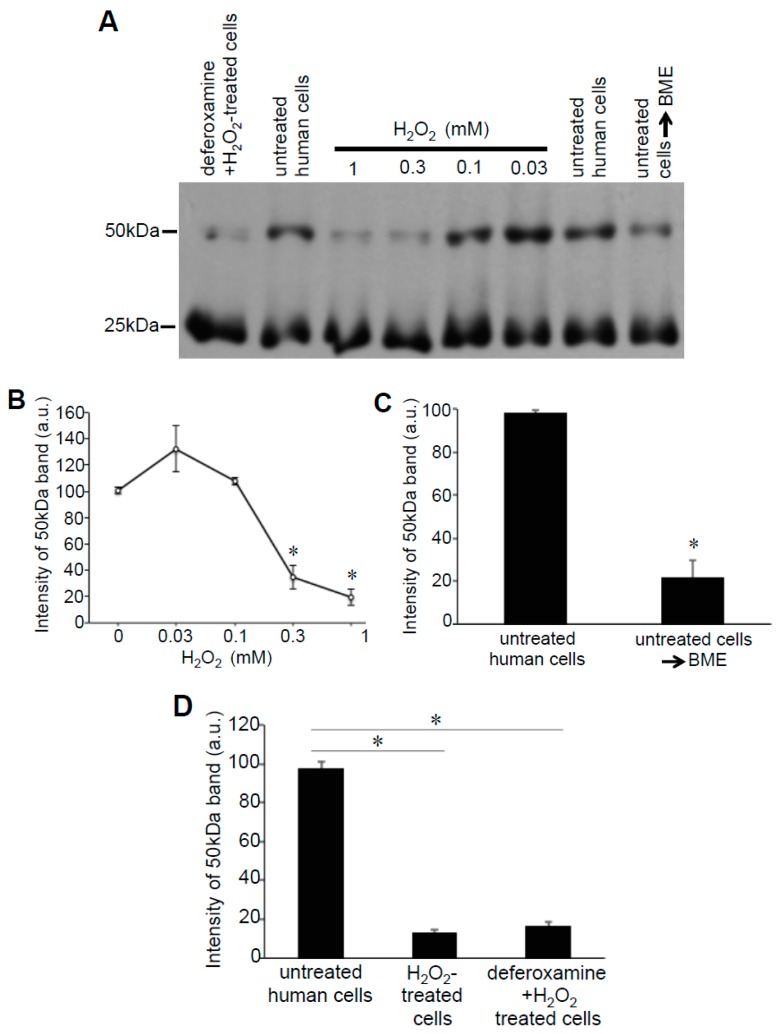
Effects of H_2_O_2_ on Prx6 multimers in human cells. (**A**) Human pulmonary artery smooth muscle cells were pre-treated with or without 50 μM deferoxamine for 30 min and then treated with or without H_2_O_2_ for 15 min. Cell lysates were prepared and subjected to SDS-PAGE with or without 5% (*v/v*) BME in Laemmli buffer, and immunoblotted with the Prx6 antibody. No Protein-SHifters were added in these experiments. (**B**–**D**) Graphs represent the means ± SEM of the intensity of the 50-kDa band (*n* = 3–6). The symbol * represents the value significantly different from the untreated control value at *p* < 0.05.

**Figure 7:**
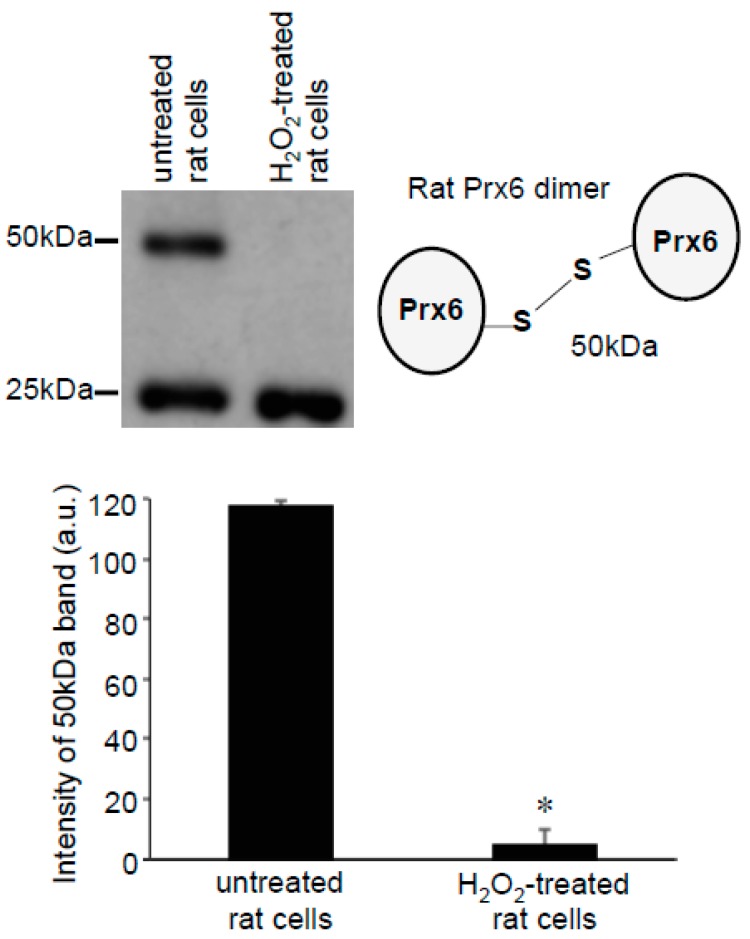
Effects of H_2_O_2_ on Prx6 dimers in rat cells. Rat pulmonary artery smooth muscle cells were treated with H_2_O_2_ (200 μM) for 15 min. Cell lysates were prepared and subjected to SDS-PAGE and immunoblotting with the Prx6 antibody. No Protein-SHifters were added in these experiments. The bar graph represents the means ± SEM of the intensity of the 50-kDa band (*n* = 3). The symbol * represents the value significantly different from the untreated control value at *p* < 0.05.

These Prx6 multimers were also detected in samples without the addition of Protein-SHifter. Figure 6A,B show that, even without Protein-SHifter, the 50 kDa dimer formation of Prx6 decreased in response to the H_2_O_2_ treatment of human cells in a dose-dependent fashion. The inclusion of β-mercaptoethanol (BME) in the loading buffer for gel electrophoresis also decreased the 50-kDa band (Figure 6A,C), confirming that the 50-kDa species resulted from protein–protein interactions through disulfide bonds. An iron chelator, deferoxamine, had no effects (Figure 6A,D), suggesting that the H_2_O_2_-induced multimer formation is not dependent on the Fenton reaction or hydroxyl radicals. A redox cycling reactive oxygen generator, 1,4-naphthoquinone, did not mimic the H_2_O_2_ effects (data not shown). In untreated rat cells, we also detected the Prx6 dimers. Figure 7 shows 25- and 50-kDa bands, corresponding to the monomer and the dimer of rat Prx6. Treatment of the cells with H_2_O_2_ (200 μM) for 15 min resulted in the elimination of 50 kDa dimers (Figure 7).

**Figure 8:**
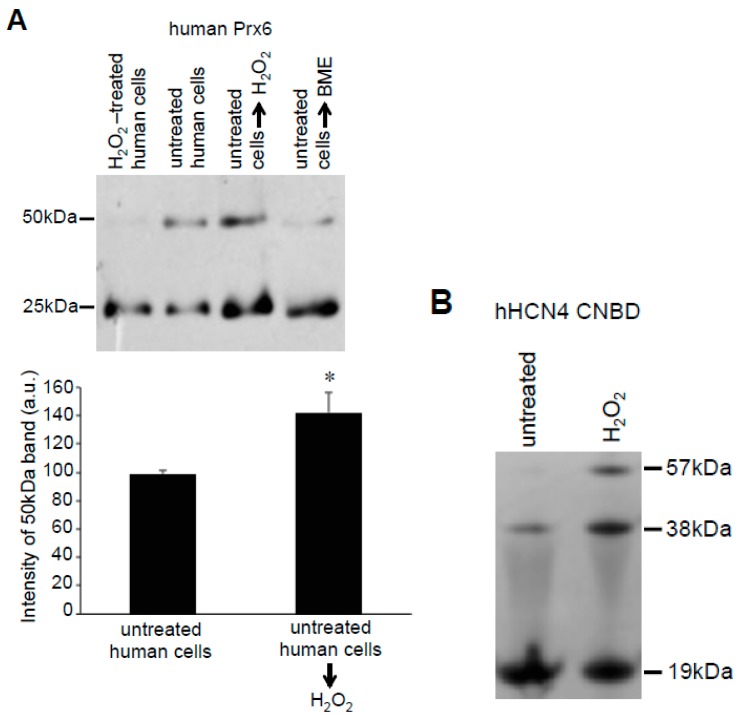
Effects of H_2_O_2_ on the isolated proteins. (**A**) Cell lysates were prepared from untreated human pulmonary artery smooth muscle cells (Lane 2) or cells treated with H_2_O_2_ for 15 min (Lane 1). Lysate proteins from untreated cells were denatured with SDS and incubated with H_2_O_2_ (Lane 3) or BME (Lane 4) in a test tube for 15 min. Protein-SHifter Plus was then added and the samples subjected to SDS-PAGE and immunoblotted with the Prx6 antibody. The bar graph represents means ± SEM of the intensity of the 50-kDa band (*n* = 3). The symbol * represents the value significantly different from the untreated control value at *p* < 0.05. (**B**) The purified recombinant CNBD domain of the hHCN4 channel was treated with H_2_O_2_ (1 mM) for 15 min and subjected to SDS-PAGE followed by Coomassie Blue staining. No Protein-SHifters were used in these experiments.

In contrast to the effects of H_2_O_2_ that occur in cells, the treatment of human cell lysates with H_2_O_2_ resulted in the increased 50-kDa Prx6 dimer formation (Figure 8A). The dimer formation decreased in the presence of BME, suggesting that dimer formation is mediated by disulfide bonds (Figure 8A). To test if this effect could also be observed in other proteins, we used the purified CNBD domain of hHCN4 channels. Consistent with the multimeric assembly of the intact HCN channels and previous studies of the isolated CNBD domains [16], non-reducing gels showed the formation of CNBD dimers (Figure 8B). Similar to the cell lysate studies of Prx6, the treatment of the purified CNBD domain with H_2_O_2_ increased the formation of 38-kDa dimers and 57-kDa trimers (Figure 8B).

## 4. Discussion

Here, we used the *-SulfoBiotics-* Protein Redox State Monitoring Kit to report on the cysteine redox state of two structurally and functionally unrelated proteins, Prx6 and CNBD of hHCN4 channels.

To our knowledge, there are currently no other methods that can easily detect thiol status, especially the number of reduced cysteine residues, of a given protein in the complex biological samples. The -*SulfoBiotics*- Protein Redox State Monitoring Kit Plus allowed us to easily assess the protein redox states in the cells as defined by the biological system, without the necessity of enriching (purifying) the protein of interest. Although there are remarkable advances in the methods of protein cysteine state detection, as summarized in the excellent reviews by Thamsen and Jakob, and Yang et al. [21,22], the methods that can easily detect thiol status and the number of reduced cysteine residues for a given protein in the complex biological samples are limited. For example, DTNB is frequently used for the detection of reduced cysteines [8]. In the biological samples, DTNB would label all reduced cysteines in the sample. Since the molecular weight of DTNB is ~400 Da, labeling of cysteines with DTNB does not introduce a significant enough shift in the molecular weight to be easily detected with Western blot using antibodies for the protein of interest. One could purify the protein of interest after the cell treatment, then measure thiol status by various methods. However, these procedures are cumbersome and the thiol status of the protein could be altered during the protein purification process. Another frequently used method is a biotin switch assay, where the free cysteines are first alkylated with methyl methanethiosulfonate, then the oxidative thiol modifications are reduced and switched for biotin [23]. To determine the cysteine status of a given protein, the proteins with biotinylated cysteines have to be purified with avidin-affinity chromatography and the protein of interest has to be visualized with the specific antibody. Therefore, an additional purification step has to be involved. Perhaps the most precise method for determining the cysteine status of a given protein is the isotope coded affinity tag (ICAT) technology that involves labeling of the accessible cysteines with the thiol-trapping reagent iodoacetamide and biotin affinity tag, and then high pressure liquid chromatography-based protein purification, followed by mass spectrometry identification of the precise protein mass [24]. Although ICAT can provide valuable and precise information on the state of cysteines in a given protein, it is much more difficult to implement than the use of the -*SulfoBiotics*- Protein Redox State Monitoring Kit Plus described here. Thus, we believe that the -*SulfoBiotics*- Protein Redox State Monitoring Kit Plus that relies on novel SHifter use is revolutionary technology that should gain attention from the research community.

While studying the redox states of cysteine residues using the *-SulfoBiotics-* Protein Redox State Monitoring Kit, we encountered novel redox events. We found that in the cells, Prx6 proteins are subjected to a very complex situation where free cysteine residues are largely in the reduced state, but some of them are oxidized forming disulfide bonds. Remarkably, the exposure to H_2_O_2_ resulted in further complex redox events including the oxidation of a specific cysteine residue without influencing others and the elimination of disulfide bonds that form multimers.

Our results indicate that, in the cellular environment, human Prx6 may be folded in a way that non-conserved Cys91 is not subjected to H_2_O_2_-dependent oxidation. Our conclusion is based on our experiments on rat Prx6, where catalytic Cys47 is the sole cysteine. This cysteine was found to be oxidized by H_2_O_2_ in rat cells under the conditions identical to experiments using human cells. Such a conclusion was further supported by our observations that the exposure of denatured human cellular proteins to H_2_O_2_ resulted in the oxidation of both cysteines in human Prx6. It is still unclear how Cys91 can stay reduced and yet inaccessible to H_2_O_2_. Further work is needed to address the issue of H_2_O_2_ accessibility to protein thiols. In this regard, the -*SulfoBiotics*- Protein Redox State Monitoring Kit and the -*SulfoBiotics*- Protein Redox State Monitoring Kit Plus should serve as useful tools for such investigations.

It is intriguing that H_2_O_2_ seems to be able to disrupt disulfide bonds in both human and rat smooth muscle cells. While it is known that H_2_O_2_ can be both an oxidant and a reductant depending on the pH, it typically triggers the formation of disulfide bonds at physiological pH [25,26]. Consistently, our experiments using two unrelated proteins, Prx6 and hHCN4 CNBD, demonstrated that the treatment of denatured proteins with the same H_2_O_2_ reagent as that used in our studies using living cells indeed promoted H_2_O_2_-induced oxidation and disulfide formation. Therefore, we propose that there are cellular mechanisms that allow for H_2_O_2_ to exhibit its disruptive capacity toward disulfide bonds. Disulfide bonds may be dynamically oxidized and reduced in the cells and, when in a reduced state, H_2_O_2_ may oxidize the thiols to alternative states, thus preventing the reformation of disulfide bonds. Alternatively, H_2_O_2_ may further oxidize disulfides to thiosulfinates and thiosulfonates [27]. Further work is needed to define the mechanism of this event.

## 5. Conclusions

In summary, the -*SulfoBiotics*- Protein Redox State Monitoring Kit and the -*SulfoBiotics*- Protein Redox State Monitoring Kit Plus allowed us to provide new information concerning protein thiol biology in the present study. It is likely that new important information will be generated using these tools in the future.
